# Retention of doctors in emergency medicine: a scoping review of the academic literature

**DOI:** 10.1136/emermed-2020-210450

**Published:** 2021-06-03

**Authors:** Daniel Darbyshire, Liz Brewster, Rachel Isba, Richard Body, Usama Basit, Dawn Goodwin

**Affiliations:** 1 Health Innovation One, Lancaster University Lancaster Medical School, Lancaster, UK; 2 Emergency Department, Salford Royal Hospitals NHS Trust, Salford, UK; 3 Paediatric Emergency Department, North Manchester General Hospital, Manchester, UK; 4 Division of Cardiovascular Sciences, The University of Manchester, Manchester, UK; 5 Emergency Department, Manchester University NHS Foundation Trust, Manchester, UK; 6 Department of Accident and Emergency, Ipswich Hospital NHS Trust, Colchester, Essex, UK

**Keywords:** emergency care systems, emergency departments, emergency department, management, HR management, training

## Abstract

**Introduction:**

Workforce issues prevail across healthcare; in emergency medicine (EM), previous work improved retention, but the staffing problem changed rather than improved. More experienced doctors provide higher quality and more cost-effective care, and turnover of these physicians is expensive. Research focusing on staff retention is an urgent priority.

**Methods:**

This study is a scoping review of the academic literature relating to the retention of doctors in EM and describes current evidence about sustainable careers (focusing on factors influencing retention), as well as interventions to improve retention. The established and rigorous JBI scoping review methodology was followed. The data sources searched were MEDLINE, Embase, Cochrane, HMIC and PsycINFO, with papers published up to April 2020 included. Broad eligibility criteria were used to identify papers about retention or related terms, including turnover, sustainability, exodus, intention to quit and attrition, whose population included emergency physicians within the setting of the ED. Papers which solely measured the rate of one of these concepts were excluded.

**Results:**

Eighteen papers met the inclusion criteria. Multiple factors were identified as linked with retention, including perceptions about teamwork, excessive workloads, working conditions, errors, teaching and education, portfolio careers, physical and emotional strain, stress, burnout, debt, income, work–life balance and antisocial working patterns. Definitions of key terms were used inconsistently. No factors clearly dominated; studies of correlation between factors were common. There were minimal research reporting interventions.

**Conclusion:**

Many factors have been linked to retention of doctors in EM, but the research lacks an appreciation of the complexity inherent in career decision-making. A broad approach, addressing multiple factors rather than focusing on single factors, may prove more informative.

**Key messages**

**What is already known on this subject**

- Addressing the retention of emergency physicians has been identified as a high priority for research in emergency medicine (EM).
- More experienced clinicians provide higher-quality and more cost-effective care. Keeping them in the specialty is vital now that efforts to improve recruitment have yielded positive results.

**What this study adds**

- Dozens of factors that influence retention have been identified in the literature.
- There is a real lack of studies looking at ways to improve retention in EM.
- Future work should address complexity; understanding the multiple interacting factors associated with retention is more likely to be beneficial than replicating correlational studies.

## Introduction

In 2012, an editorial summarising the finding of an interim report from the emergency medicine (EM) task force (a multiprofessional group set-up by the UK government’s Department of Health to address workforce issue in EM) emphatically outlined the problem:

‘Speak it loudly and speak it clearly: the specialty of Emergency Medicine (EM) in the UK has a medical staffing crisis’.^1^

In the intervening years, many of the recommendations contained in the report have been instigated. Consultant numbers have increased across the UK.^2^ Recruitment to EM training has remained consistently above 85%.^1 3^ An alternative route into EM training has been developed.^4^ The number of clinical nurse specialists, advanced practitioners and physician associates within the ED has been expanded.^4^ Despite these successes, growth in ED attendances continues to outstrip that of the workforce.^2^ Problems with attrition from training programmes^5^ and exodus of established clinicians via early retirement^6^ mean that the workforce crisis may have changed, but it certainly has not been solved. Nor is it a problem unique to the UK. The landscape may be different, but staffing EDs is a problem worldwide.^7–11^ Similar stories can be told across other specialties: psychiatry^12 13^; paediatrics^14^ and general practice^15 16^ are examples. This issue also affects emergency nurses, the largest group working in EDs.^17^

Facilitating long and productive careers, be it for emergency physicians (EPs) or the other equally vital staff groups, is of paramount importance to sustainable long-term staffing of EDs. While perhaps self-evident, it is becoming increasingly apparent that more experienced clinicians provide higher quality and better value care for their patients.^18–20^ Much of the previous work related to EM careers has focused on reasons for leaving, with the literature on burnout, the challenging working environment and the impact of out-of-hours working continuing to develop. There is, therefore, a clear gap in the literature to view this problem from an alternative perspective: not to look at why people *leave* but to focus on why those who *stay* do so, despite the universal challenges. This review is part of a programme of work focused on addressing that gap,^21^ one of the key problems facing the specialty of EM.^22^

This review is framed in terms of retention, but the terms used in academic and policy documents are inconsistent and lack clarity. The different definitions of retention, expanded on in table 1, relate to efforts by,^23–25^ or the structure of,^25^ the employing organisation to keep staff, or the proportion of workers still with an organisation after a period of time.^26–28^ We use the term retention in reference to its dictionary definition. The Merriam-Webster dictionary has three descriptions which, when taken as a whole, clarify the meaning of the term ‘retention’ without positioning it too tightly within a specific academic domain.^29^ These definitions are ‘the act of retaining’, ‘the power of retaining’ and ‘something retained’.^29^ Retention therefore is something that can be done, can be done in a particular way and has been done. This brings us to ‘retain’, which is the transitive verb to the noun of retention. The definition ‘to keep in possession or use’ is helpful as it refers to both place and action.^29^ Our use of the term is not time specific, but we recognise that in certain contexts, where measurement is important, a more technical definition may be required.

**Table 1:** Definitions of retention from the limited number of sources which define the term

Source and title	Definition
**Retention**	
Human resource management textbook: *Managing Employee Retention: A Strategic Accountability Approach*	‘the percentage of employees remaining in the organization. High levels of retention are desired in most job groups’^26^
Research in nursing and health: *The Nursing Practice Environment, Staff Retention, and Quality of Care*	‘the proportion of full-time staff nurses employed on a unit at the beginning of the study and remaining on the unit at the end of a 1-year period’^27^
**Employee retention**	
Human resource planning: *The Race for Talent: Retaining and Engaging Workers in the 21st Century*	‘the effort by an employer to keep desirable workers in order to meet business objectives’^23^
*International Journal of Advance Research in Computer Science and Management Studies*: Review paper—study on employee retention and commitment	‘a technique adopted by businesses to maintain an effective workforce and at the same time meet operational requirements’^24^
*Journal of Economics, Management and Trade*: Human resource management practices and employee retention: a review of literature	‘the hierarchical arrangements and practices utilised as a part of the organisation to keep key workers from leaving the association’^25^
**Volunteer retention**	
Independent research organisation report: *Volunteer Management Practices and Retention of Volunteers*	The percentage of volunteers involved with the organisation 1 year ago who are still involved today.^28^

As well as retention, the literature contains a myriad of other terms which overlap in stated definition and usage with many being used interchangeably. For example, the word ‘attrition’ was frequently used interchangeably with the terms ‘dropouts’, ‘turnover’, ‘brain drain’, ‘losses’, ‘premature departure’ and ‘separation’.^30^ The commonly used terms for both staying in a role or leaving it are defined in table 2.

**Table 2:** Definitions for terms related to retention

Terms	Definition
**Terms related to staying in a role**	
Sustainable careers	‘the sequence of an individual’s different career experiences, reflected through a variety of patterns of continuity over time, crossing several social spaces, and characterised by individual agency, herewith providing meaning to the individual’^74^
Career longevity	‘a fundamental metric that influences the overall legacy of an employee because for most individuals the measure of success is intrinsically related, although not perfectly correlated, to his or her career length’^75^
Employee/personnel loyalty	‘may be measured in terms of expressed commitment to the (organisation) and its mission and in terms of length of employment’^76^
Organisational commitment	‘the relative strength of an individual’s identification with and involvement in a particular organization’^77^
Occupational embeddedness	‘the totality of forces that keep people in their present occupations’^78^
**Term related to leaving a role**	
Turnover	‘unplanned loss of workers who voluntarily leave and whom employers would prefer to keep’^23^
Intention to quit	‘how often the respondents seriously considered quitting the job, whether they wanted to quit, and whether they were actually planning to quit’^79^
Exodus	Not defined in the literature. The Cambridge Dictionary has a business English definition of ‘the movement of lots of people or things away from a place’^80^
Attrition	‘exits from the workforce’^30^ generally presented as a rate over time’
Career mobility	‘the transition from one position to another’^81^
Organisational change	‘any change in the employing firm’^78^
Job change	‘any substantial changes in work responsibilities, hierarchical levels, or titles within an organization. It includes internal promotions, transfers, and demotions.’^78^
Occupational change	‘transitions that require fundamentally new skills, routines, and work environments and require fundamentally new training, education, or vocational preparation’^78^

Because of these definitional inconsistencies, the search included a wide selection of these terms. The included papers are those that address retention, as we have defined, regardless of the terminology used by the authors.

## Methods

The protocol for this review was published in advance and is available open access.^31^ This paper focuses on the academic literature; the scoping review of the grey literature discussed in the protocol will be reported separately.

The aim of this study, aligning with the scoping review methodology,^32^ was to map the extent of the literature directly pertaining to retention of doctors in EM. More specifically, this involves identifying the types of evidence available, collating the key characteristics of papers, identifying the key definitions and concepts, and delineating and analysing the gaps in the literature. This is in keeping with the predetermined review question:

- Primary question: What is known about retention of doctors in EM?
- Subquestion 1: What factors have been studied relating to retention of doctors in EM?
- Subquestion 2: What interventions have been implemented to improve retention of doctors in EM?

A search of MEDLINE, Embase, Cochrane, HMIC and PsycINFO was initially completed on 15 March 2019 by Helen Elwell, clinical librarian at the British Medical Association Library, and then updated for papers published in the interim, on 14 April 2020 (Cochrane and MEDLINE) and 21 April (Embase, HMIC and PsycINFO). This was supplemented by searches of Business Source Complete, Proquest Business Premium Collection and Emerald Insight. The search terms for Ovid MEDLINE are available in table 3, with the remainder in online supplemental appendix 1. Reflecting the nature of scoping reviews and the research questions, this search aimed for breadth of coverage. The Preferred Reporting Items for Systematic Reviews and Meta-Analyses Extension for Scoping Reviews) checklist is included in online supplemental appendix 2.

10.1136/emermed-2020-210450.supp1 Supplementary data

10.1136/emermed-2020-210450.supp2 Supplementary data

**Table 3:** Ovid MEDLINE search strategy

	Search term
1	physicians/ or exp pediatricians/
2	(physician$ or doctor$ or trainee$ or foundation year or fy1 or fy2 or sho or shos or senior house officer$ or registrar$1 or staff grade or associate specialist$ or consultant$).mp.(mp=title, abstract, original title, name of substance word, subject heading word, floating sub-heading word, keyword heading word, organism supplementary concept word, protocol supplementary concept word, rare disease supplementary concept word, unique identifier, synonyms)
3	p?ediatrician$.mp.
4	(medical practitioner$ or clinician$).mp.
5	or/1–4
6	emergency medical services/ or emergency service, hospital/ or trauma centers/
7	emergency medicine/ or pediatric emergency medicine/
8	(emergency medical services or emergency service or trauma center$ or trauma centre$).mp.
9	(emergency medicine or pediatric emergency medicine).mp.
10	(emergency department$ or emergency room or casualty department$ or “A&E”).mp.
11	“accident and emergency”.mp.
12	emergency training program$.mp.
13	emergency medical care.mp.
14	or/6–13
15	5 and 14
16	workforce/ or health workforce/ or personnel loyalty/ or work schedule tolerance/ or work-life balance/ or workload/ or personnel turnover/
17	burnout, psychological/ or burnout, professional/ or exp occupational stress/
18	Career Choice/
19	career mobility/
20	(workforce or manpower or staffing or retention or work-life balance or turnover or leaving medicine or exiting or burnout).mp.
21	(career adj4 (choice or mobility or progress$ or ladder or promotion or advancement or satisfaction)).mp.
22	or/16–21
23	15 and 22

**Figure 1:**
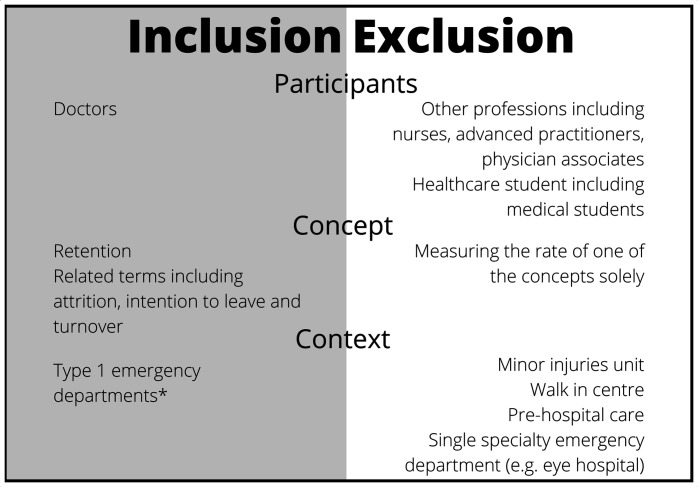
Inclusion and exclusion criteria. *Type 1 EDs are ‘consultant led 24 hour service with full resuscitation facilities and designated accommodation for the reception of accident and emergency patients’.^82^

**Figure 2:**
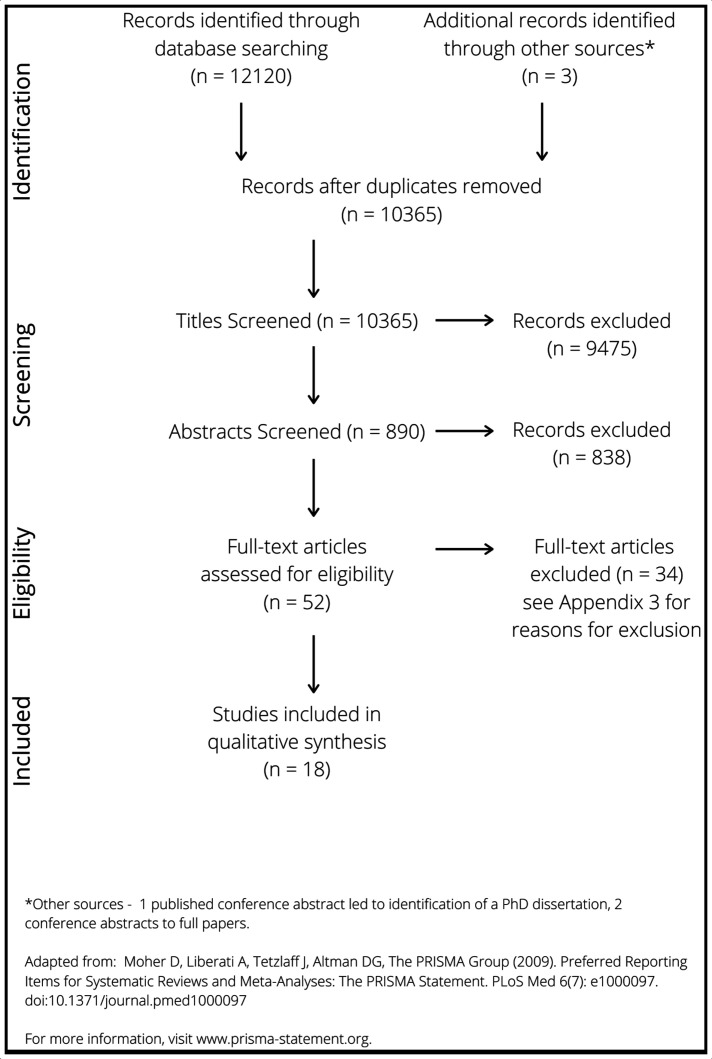
Preferred Reporting Items for Systematic Reviews and Meta-Analyses diagram.

All searches were limited to English language. No date limitations were applied. Given the vast number of results, a team-based multistage approach was undertaken. Titles were reviewed by DD and clearly irrelevant items were excluded. Abstracts were then independently reviewed by DD and UB. To ensure consistency, this was piloted with tranches of 20 until complete adherence was achieved and reviewers were in frequent communication during the abstract screening process. Abstracts were reviewed against the inclusion criteria (see figure 1 and the protocol^31^), with those clearly not meeting the criteria excluded. Full-text articles were then accessed and again compared with the inclusion criteria; see figure 1 for inclusion and exclusion criteria and figure 2 for the Preferred Reporting Items for Systematic Review and Meta-Analysis Protocols (PRISMA) diagram.

The following data were extracted from the included articles: citation, methodology, factors influencing retention, efforts to improve retention and other findings relevant to retention of doctors in EM. Data are presented to summarise the different approaches to doctor retention in EM that are represented in the literature and to give a picture of where the gaps in the literature lie. Papers not pertaining to EPs and those that did not go beyond measuring a rate of retention (or attrition) were excluded. This is because defining the rate of retention in EM is not a research question for this study and would be best answered with a complementary methodology, such as systematic review and meta-analysis.

## Results

The result of database searching is presented in the PRISMA diagram (see figure 2). The studies excluded at the eligibility (full-text reading) stage, including rationale for exclusion, are summarised in online supplemental appendix 3. Methodological details and study characteristics of included papers are available in online supplemental appendix 4. A brief summary of each paper alongside the links to the research questions is in table 4.

10.1136/emermed-2020-210450.supp3 Supplementary data

10.1136/emermed-2020-210450.supp4 Supplementary data

**Table 4:** Summary of included papers

Paper details		Extraction			
Author, year and origin	Journal and type of paper	Method and aim of study	Factors influencing retention	Efforts to improve retention	Other relevant findings
Estryn-Behar *et al.*,^48^ 2011, France	*Emergency Medicine Journal*, research paper	Questionnaire using several psychological scales applied to 538 EPs and 1924 matched physicians from other specialties, aimed to measure and correlate aspects of working life and intention to leave.	Intention to leave linked with quality of teamwork, burnout, musculoskeletal disorders, job offers from outside medicine, absence of continuing education for 12 months, worry about mistakes, harassment by superiors, lack of influence at work and tense relations with administration.	None.	Working conditions may be more important than pay.
Feitosa-Filho *et al.*,^49^ 2017, Brazil	*Revista da Associação Médica Brasileira*, research paper	Questionnaire of 659 ED physicians across a region of Brazil assessing workplace characteristics, EPs training, main reason for working in EM, work satisfaction and reasons why they might leave; primary aim to quantify work characteristics.	Higher job satisfaction correlated with lower intention to quit.	None.	81.3% said they intended to stop working at the ED in the next 15 years, pointing out ‘excessive stress at work’ as their main reason.
Fitzgerald *et al.*,^40^ 2017, UK	*Emergency Medicine Journal,* research paper	Interpretive phenomenological analysis study based on 18 semistructured interviews with EM consultants in southwest England, primary aim to explore the experience of psychological distress and well-being.	Consultants perceive the physical and emotional strain of EM work to be unsustainable, peer social support and developing new roles can help sustainability.	The emergence of self-care and compassion dialogues may be beneficial.	Participants unanimously identified with the term ‘sustainability’ when describing their emotional and physical status.
Goldberg *et al.*,^45^ 1996, USA	*Academic Emergency Medicine*, research paper	Questionnaire of 1272 attendees at an EM conference over 4 years; questionnaire incorporated the Maslach Burnout Inventory and practice demographics, including intent to practice EM in the future, aiming to measure burnout and to identify predictive factors.	Intention to leave EM correlates with a higher burnout score.	None.	None.
Hall *et al.*,^33^ 1992, USA	*Academic Emergency Medicine,* research paper	Postal questionnaire sent to US EPs who finished training between 1978 and 1982; 539 responses; compared practice characteristics of those who still practice EM with those who have left.	Those who left were less likely to be board certified in EM, more likely to be board certified in another specialty, were less likely to work with residents and reported lower income.	None.	None.
Hall and Wakeman,^36^1999, USA	*The Journal of Emergency Medicine*, research paper	Questionnaire sent to residency trained EPs about demographics, work characteristics, attrition and reasons for leaving. 1638 responses. Aims to measure practice characteristics, how careers change with time and career longevity.	EPs with higher income had lower attrition, but those who left did not rate income as a reason for leaving. EPs who had done a residency or fellowship outside EM or were not board certified had higher attrition.	None.	Clinicians decreased clinical work and increased other work though their career.
Holmes,^47^ 2019, Australia	*Emergency Medicine Australasia*, brief communication	Discussion paper (termed ‘Perspective’ in this journal) giving the authors view on sustainable careers in EM is Australia. Two areas of focus are burnout and the ageing EP.	The author believes that credentialing in a subspecialty field, maintaining professional links and lifelong learning may help sustainability.	Unreferenced claim that some countries do not require older doctors to work on-calls or out of hours and a belief that this would help in Australia.	The authors state that 'there has been insufficient recognition of the particular needs of older physicians, including that they tolerate shift work and night duty more poorly than their junior colleagues.'
James and Gerrard,^39^ 2017, UK	*Emergency Medicine Journal*, research paper	Semistructured interviews with 10 consultants from Welsh EDs exploring what attracted them to the career and what keeps them there.	Diagnostic challenges, teaching junior colleagues, teamwork, flattened hierarchy, flexible working and positive work–life balance.	Participants thought that improving flow and staffing would help retention.	None.
Kalynych,^41^ 2010, USA	UNF Graduate Theses and Dissertations, dissertation, primary research	Questionnaire of 273 EM residents measuring margin in life (psychological theory of adult development) scale and intention to leave; aim to assess for a difference between EM residents scores and remediation, intention to quit and actual attrition.	No correlations identified.	None.	None.
Lloyd *et al.*,^35^ 1998, Canada	*Academic Emergency Medicine*, research paper	Questionnaire to compare two different job satisfaction instruments with 14 'reasons for leaving'. The study aim was to evaluate the predictive validity of the Emergency Physician Job Satisfaction and Global Job Satisfaction instruments.	A low Global Job Satisfaction instrument score is associated with leaving EM (the test characteristics mean it is not a useful predictor).	Scheduling, as an extrinsic component of job satisfaction, is amenable to change.	Ranked reasons for leaving EM and compared with a previous (US) cohort.
Mallon,^44^ 2000, USA	*The Journal of Emergency Medicine*, letter	Letter commenting on Hall and Wakeman 1999 and referencing the authors’ study (Goldberg *et al.* 45)	Reiterates key points from Goldberg *et al.* 45	None.	Concern about overestimating attrition and oversubscribing the workforce devaluing EPs and creating job insecurity.
Murphy,^46^ 2014, Ireland	*Irish Medical Journal*, editorial	Editorial outlining the retention problem in Ireland.	Better support and less stress, personal development.	Streamline training, ministerial review of the medical workforce.	There is a retention problem across Irish medicine; it is more visible in EM.
Pflipsen *et al.*,^37^ 2019, Ireland	*Irish Journal of Medical Science*, research paper	Questionnaire sent to those who had left the Irish EM training scheme. 30 respondents, aim to gain insight into reasons for attrition from EM training in Ireland	Lack of training and supervision negatively impacted retention, as do excessive workloads and poor working conditions.	None.	Findings similar in other specialties in Ireland.
Smith and Dasan,^42^ 2018, UK	*British Journal of Hospital Medicine (London)*, review	Pragmatic review of academic and policy literature aiming to describe the impact increasing working pressure is having on staff in the ED and to begin to explore the potential for developing sustainability within the workforce.	Occupational stress and burnout negatively impact retention.	Job planning, less than full time working, portfolio careers, appropriate renumeration, well-being; introduces sustainability work from Royal College of Emergency Medicine.	None.
Takakuwa *et al.*,^50^ 2013, USA	*Academic Medicine*, research paper	Questionnaire sent to leads of EM training programmes, 78 responses, aims to describe the policies, practices and attitudes of EM leaders about workforce issues, particularly for EPs in the last decade of their career.	A strategic approach to staffing overnight shifts, various different policies inconsistently applied.	Refers to documents related to ageing and EM work produced by the group that did this research.	Variable and inconsistent approach to the role of the EP in the final 10–15 years of their career.
Xu *et al.*,^34^ 1994, USA	*Academic Emergency Medicin*e, research paper	Cohort Study using routinely collected data; looking at three groups: those who choose EM and stay, those who move into EM and those who leave; compares academic performance, age and indebtedness with an aim to identify factors that may have contributed to career change.	High academic performance and high indebtedness are factors associated with choosing or staying in EM.	None.	Indebtedness is complex.
Xu and Veloski,^38^ 1991, USA	*Academic Medicine,* brief communication	Questionnaire sent to graduates of a specific university who had chosen EM at graduation, 36 responses, aim to measure factors influencing their decision to continue EM careers.	Most important factors for remaining in EM were challenging diagnostic problems, predictable working hours, intellectual content of the specialty and income.	None.	Educational debt a minor factor.
Zun *et al.*,^43^ 1988, USA	*American Journal of Emergency Medicine*, discussion	Case report and literature review. Describes a case where a patient dies after being discharged from the ED. This leads to a discussion, citing relevant literature, about the stress such an event causes and stress for EPs in general.	Discusses factors that lead to stress for EPs, specifically errors, incivility by colleagues and working patterns.	Authors’ thoughts; open discussion as key to helping manage stress, also helped by time management systems, lifestyle approaches and specific relaxation approaches.	None.

EP, emergency physician.

### Factors influencing retention

The identified papers explored retention in different ways. Three papers analysed factors that correlated with intention to leave, five correlated with reasons for having quit or attrition rates,^33–37^ two with reasons for continuing EM work^38 39^ and in one case with reasons why EPs might leave or why they stay.^40^ Two of the papers found no practically applicable correlations: margin in life (a psychological theory of adult development) scale had no correlation with intention to quit,^41^ and while a low score on the Global Job Satisfaction instrument scale was correlated with leaving EM, its test characteristics meant that it was not a useful predictor of quitting.^35^ A pragmatic literature review described elements of EM that negatively impacted retention^42^; a case report and discussion explored incidents that may lead an EP to quit^43^; a letter^44^ commenting on an included study^36^ mainly reiterated points from the letter writers own study (also included in this review), which correlated burnout with intention to quit.^45^ The two remaining letters focused on sustainable careers in Ireland and Australia, respectively.^46 47^ The diversity of approaches used in the articles included in the review has led to a large number of different factors correlating with retention in EM; these are documented in table 5.

**Table 5:** Items related to retention, attrition or intention to leave EM

Experience of work	Lack of quality teamwork^48^ Teamworking and non-hierarchical structure^39^ Harassment by supervisors^48^ and incivility^43^ Job satisfaction^35 49^ Excessive workloads^37^ Poor working condition^37^ Peer support^40 46^ and professional links^47^ Diagnostic challenges^38 39^ Errors^43^ Lack of influence at work^48^
Training and education	Absence of continuing professional education^48^ Lifelong learning^46 47^ Lack of training and supervision^37^ Board certification (higher training) in EM^33 36^ Board certification in another specialty^33 36^ Fellowship in another specialty^36^ Work with trainees^33^ Teaching^39^ New roles^40^ Subspecialty training^47^
Impact of work	Worry about mistakes^48^ Musculoskeletal complaints^48^ Physical and emotional strain^40^ Burnout^42 45 46 48^ Occupational stress^42^ Stress^46^
Work–life balance	Debt^34^ Income^33 36 38^ Flexible working^39^ and predictable hours^38^ Strategic approach to shift work^50^ Antisocial working patterns^43^ Receiving a job offer outside of medicine^48^

EM, emergency medicine.

### Efforts to influence retention

The majority of papers did not directly address efforts to improve retention.^33 34 36–38 41 44 45 48 49^ Of those that did, only three drew conclusions from empirical work.^35 39 40^ The participants in James and Gerrard’s study said that improving flow and staffing would improve retention,^39^ while those in the study from Fitzgerald *et al.* thought that the emergence of self-care and compassion dialogues may be beneficial.^40^ Lloyd *et al.* stated that work scheduling (rostering), as an extrinsic part of job satisfaction, is amenable to change and therefore has potential to improve retention.^35^ However, it should be noted that in this study, evaluating the predictive validity of two job satisfaction scales, while one of the scales they tested had a statistically significant correlation with attrition, we found that the test characteristics of this relationship mean it lacks predictive utility.

The other studies offered suggestions from a range of perspectives. One paper referenced documents on ageing and the EM workforce,^50^ while another offered an unreferenced statement that in some countries, ‘older doctors are not required to participate in after-hour rosters’.^47^ An editorial explained that streamlining training and a ministerial review of the broader medical workforce in Ireland, both ongoing when published in 2014, might help retention.^46^ The case report and discussion paper provided examples and references for stress management techniques that are relevant to EPs, which the authors postulated might help career sustainability.^43^ Smith and Dasan’s review paper highlighted measures to improve retention, reflecting some of the work of the previous section, specifically job planning, less than full time working, portfolio careers, appropriate remuneration and well-being.^42^ They then introduced sustainability work from the UK’s Royal College of Emergency Medicine (RCEM).

### Other findings related to retention

The participants in the study by Fitzgerald *et al.* universally identified with the term ‘sustainability’ when discussing their emotional and physical status related to their work.^40^ This parallels the terminology used in the review by Smith and Dasan, also from the UK, along with the materials from RCEM that it references.^42^

In their study of residency trained US EPs, Hall and Wakeman found that clinicians tended to decrease clinical work and increase other types of work, such as teaching and administration, as their careers progressed.^36^ Takakuwa *et al.* found that policies related to ageing were inconsistent for the EPs approaching the final years of their career.^50^ While both studies were from the USA, this message is mirrored in Holmes’ opinion piece from Australia.^47^

In their large study of French EPs, Estryn-Behar *et al.* found that working conditions may be more important than pay.^48^ Related to money, Xu and Veloski^38^ and Xu *et al* 34 found that having educational debt was associated with staying in EM in the USA.

A study from Brazil by Feitosa-Filho *et al.* found that 81.3% of EPs planned to stop working in the ED in the following 15 years.^49^ A letter by Mallon (from the USA),^44^ commenting on Hall and Wakeman (again from the USA),^36^ expressed concern about the possibility of overestimating attrition in the USA, leading to having too many trained EPs, leading in turn to job insecurity and a fall in the perceived value of EPs.

## Discussion

It has been over 20 years since the first paper on retention of EPs, identified by this review, was published,^43^ with a seeming trend of increased activity in this domain reflecting the growth of EM research globally.^51^ Despite this relative growth, the absolute number of papers is low, and those that have been produced display significant methodological heterogeneity. The most frequently used methodological approach has been measurement, using a pre-existing scale of a psychological construct and testing to see if it is correlated with retention (or a term related to it).^39 41 45 48^ Burnout is the most assessed construct,^45 48^ reflecting the prominence of burnout research in both the EM^52 53^ and wider medical literature.^54–56^ Again, reflecting the wider medical literature on burnout, problems arise with definitions and interpretations of the term, different cut-offs used for the threshold for defining burnout, different burnout inventories used and type I errors (false positives) when multiple tests for correlation are undertaken.^57^

Despite these issues, it is useful that two studies from different continents, using two different validated measures, have linked burnout with retention (both via intention to leave),^45 48^ a finding that is replicated in the nursing profession,^58^ teachers^59^ and volunteers.^60^ Margin in life (a psychological theory of adult development) was not correlated with intention to leave^41^—the measure is most often correlated with readiness for change such, as organisational restructuring or merger.^61 62^ While global job satisfaction was correlated with attrition, Xu *et al.* found that the correlation was not strong enough to use the scale predictively,^34^ a finding consistent with the broader human resources literature, which finds that intrinsic job satisfaction is negatively correlated to turnover, whereas extrinsic job satisfaction has no statistically convincing link.^63^

The second prominent group of studies measured aspects of work life and alongside either attrition^33 36^ or intention to leave,^49^ or described policies related to retention in the final third of an EP’s working life.^50^ Of the many aspects of work life that Feitosa-Filho *et al.* assessed, job satisfaction— measured as a single multiple choice question with the options ‘satisfied’, ‘neutral’ and ‘dissatisfied’—was the only one showing a statistically significant correlation with intention to leave.^49^ The study by Lloyd *et al.*, discussed earlier, linked job satisfaction and quitting EM but not strongly enough to offer a predictive test.^35^ Feitosa-Filho *et al.* found that 64% of their EPs who were satisfied and 94% who were dissatisfied intended to quit in the next 15 years; however, the baseline characteristics of their study from Brazil make it equally difficult to apply a different practice setting.^49^ This does not mean that job satisfaction should be discounted—there is a long history from economics marking satisfaction as a ‘major determinant of labour market mobility’^64^—and it has been linked with concepts related to retention across several professional groups, including nurses,^65^ general practitioners,^66^ physician assistants and nurse practitioners.^67^ The second aspect of work life relates to training, with board certification (postgraduate specialty examinations in the USA) and fellowships (a period, generally a year, of subspecialty training related to the primary training specialty) correlating with lower attrition.^33 36^ What it is about fellowship or board certification that influences attrition is not clear, but other studies have linked high academic achievement while at medical school^34^ and the intellectual content, specifically diagnostic challenges, of the specialty as important.^38^ These features can be threatened by a lack of training or supervision, excessive workloads and poor working conditions.^37^

Most of the studies examined retention from a broad, though necessarily superficial perspective. However, two studies took the opposite approach, gaining in-depth accounts from a relatively smaller number of participants.^39 40^ Describing the physical and emotional strain of working in the ED as ‘unsustainable’ adds credence to the idea that psychological measures (such as burnout) may have utility in efforts to improve retention while simultaneously suggesting that such measures may be an oversimplification. The more social aspects of EM, such as the flattened hierarchy^39^ and peer social support,^40^ move the discussion away from the individual approach to retention to the idea that the interactions between the people involved in the work of EM might be key.

The papers in this study support the notion that pay is linked to retention^26^ with higher income correlating with lower attrition^36^ and with those who leave the specialty having had lower incomes than those who stayed,^33^ though this finding could be skewed by salaries generally rising with career length. Income was reported as a major factor in decisions to stay in EM.^38^ Educational debt is another factor, representing a strong correlate with staying in EM in one study^34^ and a minor factor in another.^38^ It should be noted that these studies are from the USA, where both income for doctors and educational debt are significantly higher than most other counties, with the study by Estryn-Behar *et al.* from France concluding that ‘working conditions may be more important than pay’.^48^ The relationship between pay and retention is more complex than a linear correlation, so that even with high pay, ‘pay dissatisfaction can lead to turnover’.^26^ Other factors, beyond the amount of remuneration received, make pay more complex with perceptions of fairness being the most important. This is described at two levels. This first, distributive justice, refers to the distribution of pay within an organisation,^68^ while the second, procedural justice, is about the process through which pay is administered,^69^ with both repeatedly linked to retention both within^69^ and outside of healthcare.^26 68^

While some of the studies presented here discussed aspects of work that may be amenable to change, in order to improve retention, none tested this as a hypothesis directly. The lack of interventions in the academic literature may be due to them being reported elsewhere. It is highly unlikely that a change to a single aspect of work influencing retention would lead to measurable change—the required number of participants and scale of impact would likely be too large to be feasible. Moving towards recognising, studying and implementing change with complexity,^70^ rather than imposing false notions of simplicity, will be key to any successful interventions, something that the review by Smith and Dasan alludes to.^42^

The concept of career change or evolution may be more closely aligned to careers in EM than the more linear concept of promotion, demotion and resignation. Hall and Wakeman found that clinicians decreased clinical work and increased other work though their career.^36^ Portfolio careers, here meaning role diversity within a profession rather than the definition more common outside of healthcare—‘individuals develop a portfolio of skills that they sell to a range of clients’,^71^ are gaining increasing prominence in discourses about health professions careers.^42 72^ The idea that using skills developed through professional training and experience in related roles helps prevent people getting bored or jaded has strong face validity and, while there is a small body of research supporting this, the findings are not conclusive.^16^

**Figure 3:**
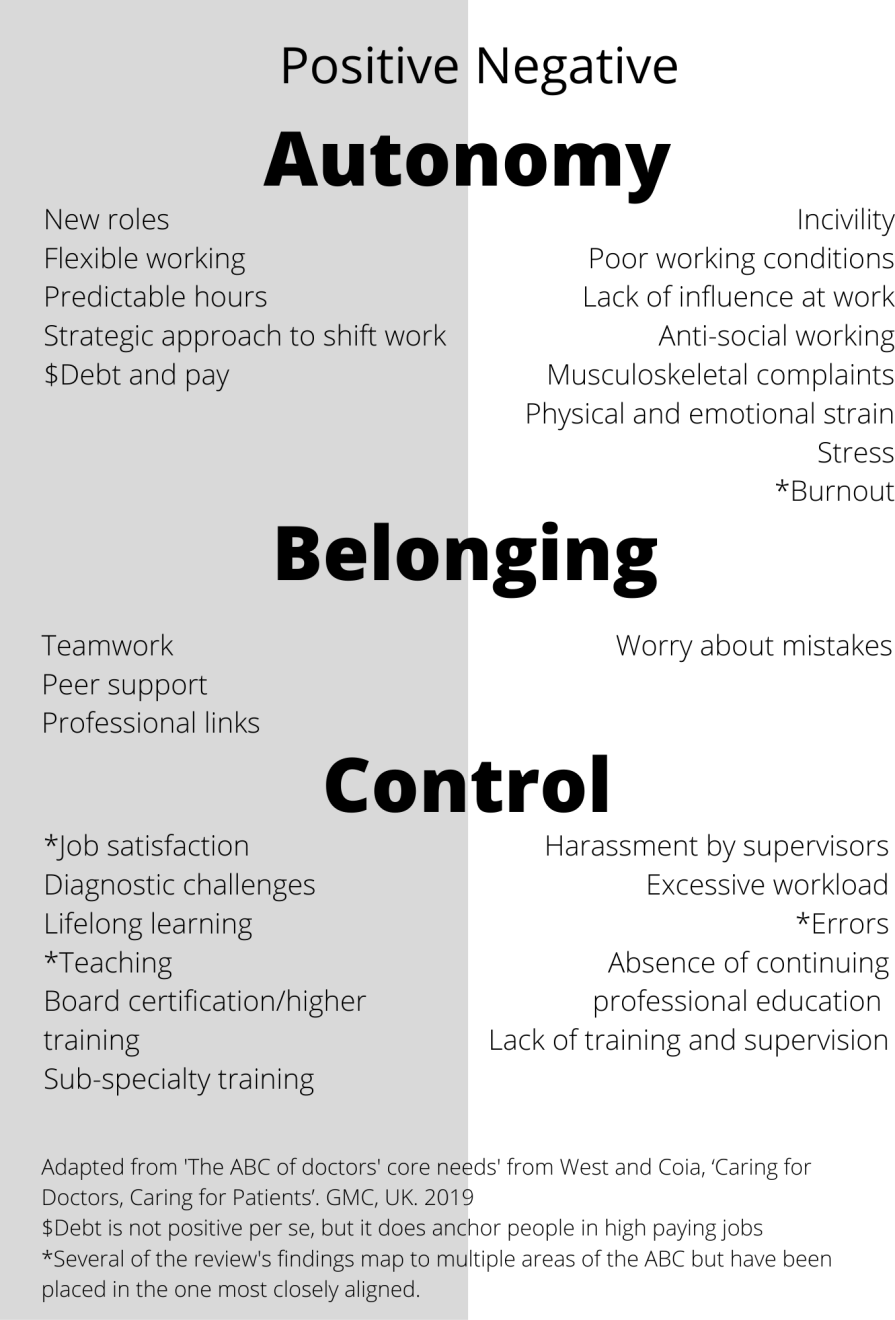
Review findings mapped to the ABC of doctors' core needs.

A recent body of work published by the UK medical regulator (General Medical Council (GMC)) started with the premise that patient safety is dependent on doctors’ well-being, integrated a summary of the existing academic literature with case studies and developed the ABCs of doctors’ core needs; the findings of this review can be mapped to the ABC structure (see figure 3). While not the primary aim of this work, it is clear that retention was within its broader remit, with the foreword from the chair of the GMC stating that ‘If we act together we will avoid losing good doctors and seize a golden opportunity to tackle the challenges the health service must meet now and in the future’.^73^

Referring to the table of definitions (table 2), we found that while the papers use terms related to retention, there is a universal lack of clear definitions; for example, Hall *et al.* use the term ‘career longevity’ without defining it.^33^ Estryn-Behar *et al.* do not define ‘intention to leave’, but they do state the question they use to measure it, and in other instances, the terms related to retention come from interview participants rather than the researcher.^39 40^ Lloyd *et al.* use an approach that avoids clearly defined terms, instead using short descriptive statements such as ‘left job and EM altogether’ consistently.^35^ However, the general lack of clarity in terms related to retention is a real weakness of this body of literature. Terms such as ‘attrition rate’, meaning different things in different papers, if indeed they are clearly defined at all, are a potential source of confusion and misinterpretation. The table of excluded papers (online supplemental appendix 3) reinforces this confusion, with papers relating to intention to leave referring to the workplace rather than the profession, and many papers presenting an estimate for levels of intention to leave or turnover, which, while useful in that specific context, does not help with developing understanding of retention.

The scoping review process has inherent limitations; we have described the factors that influence retention but not the scale of influence of each factor. The breadth of types of papers meant that several different quality appraisal tools would have been required to do this and a decision was therefore made that this would not have added significantly to the current study.

The literature related to retention of doctors in EM yielded a variety of factors with complicated and mostly unclear interactions. Interventions to improve retention have a very limited research base. Linked to the factors influencing retention, it is likely that programmes to address a single issue are unlikely to be effective; instead, holistic approaches cutting across the multiple domains of work life should be trialled. Future research needs to embrace this complexity rather than try to eradicate it.

## Data Availability

All data relevant to the study are included in the article or uploaded as supplemental information.
