# Supplementary material for: Retention of doctors in emergency medicine: a scoping review of the academic literature
Source: Emerg Med J. 2021 Jun 3;38(9):663–72. doi: 10.1136/emermed-2020-210450 (PMC8380914; doi:10.1136/emermed-2020-210450)
Supplement: Supplementary data [file emermed-2020-210450supp001.pdf]

Appendix 1 Search Strategies

OVID MEDLINE

	Search Term
1	physicians/ or exp pediatricians/
2	(physician$ or doctor$ or trainee$ or foundation year or fy1 or fy2 or sho or shos or senior house officer$ or registrar$1 or staff grade or associate specialist$ or consultant$).mp. [mp=title, abstract, original title, name of substance word, subject heading word, floating sub-heading word, keyword heading word, organism supplementary concept word, protocol supplementary concept word, rare disease supplementary concept word, unique identifier, synonyms]
3	p?ediatrician$.mp.
4	(medical practitioner$ or clinician$).mp.
5	or/1-4
6	emergency medical services/ or emergency service, hospital/ or trauma centers/
7	emergency medicine/ or pediatric emergency medicine/
8	(emergency medical services or emergency service or trauma center$ or trauma centre$).mp.
9	(emergency medicine or pediatric emergency medicine).mp.
10	(emergency department$ or emergency room or casualty department$ or "A&E").mp.
11	"accident and emergency".mp.
12	emergency training program$.mp.
13	emergency medical care.mp.
14	or/6-13
15	5 and 14
16	workforce/ or health workforce/ or personnel loyalty/ or work schedule tolerance/ or work-life balance/ or workload/ or personnel turnover/
17	burnout, psychological/ or burnout, professional/ or exp occupational stress/
18	Career Choice/
19	career mobility/
20	(workforce or manpower or staffing or retention or work-life balance or turnover or leaving medicine or exiting or burnout).mp.
21	(career adj4 (choice or mobility or progress$ or ladder or promotion or advancement or satisfaction)).mp.
22	or/16-21
23	15 and 22

EMBASE

	Search Term
1	exp physician/ or pediatrician/
2	(physician$ or doctor$ or trainee$ or foundation year or fy1 or fy2 or sho or shos or senior house officer$ or registrar$1 or staff grade or associate specialist$ or consultant$).mp. [mp=title, abstract, heading word, drug trade name, original title, device manufacturer, drug manufacturer, device trade name, keyword, floating subheading word, candidate term word]
3	p?ediatrician$.mp.
4	(medical practitioner$ or clinician$).mp.
5	or/1-4
6	emergency health service/ or hospital emergency service
7	emergency medicine/ or pediatric emergency medicine/
8	(emergency medical services or emergency service or trauma center$ or trauma centre$).mp
9	(emergency medicine or pediatric emergency medicine).mp.
10	(emergency department$ or emergency room or casualty department$ or "A&E").mp.
11	"accident and emergency".mp.
12	emergency training program$.mp.
13	emergency medical care.mp.
14	or/6-13
15	5 and 14
16	emergency physician/
17	or/15-16
18	workforce/ or health care manpower/ or work schedule/ or work-life balance/ or workload/
19	burnout/ or exp job stress/
20	Career Choice/
21	career mobility/
22	(workforce or manpower or staffing or retention or work-life balance or turnover or leaving medicine or exiting or burnout).mp
23	(career adj4 (choice or mobility or progress$ or ladder or promotion or advancement or satisfaction)).mp
24	or/18-23
25	17 and 24

Cochrane

	Search Term
1	MeSH descriptor: [Physicians] this term only
2	MeSH descriptor: [Pediatricians] explode all trees
3	Physician* or doctor* or trainee* or "foundation year" or fy1 or fy2 or sho or shos or "senior house officer*" or registrar or registrars or "staff grade" or "associate specialist*" or consultant*
4	pediatrician* or paediatrician*
5	"medical practitioner*" or clinician*
6	#1 or #2 or #3 or #4 or #5
7	MeSH descriptor: [Emergency Medical Services] this term only
8	MeSH descriptor: [Emergency Service, Hospital] this term only
9	MeSH descriptor: [Trauma Centers] this term only
10	MeSH descriptor: [Emergency Medicine] this term only
11	MeSH descriptor: [Pediatric Emergency Medicine] this term only
12	"emergency medical services" or "emergency service" or "trauma center*" or "trauma centre*"
13	"emergency medicine" or "pediatric emergency medicine"
14	"emergency department*" or "emergency room" or "casualty department*" or "A&E"
15	"accident and emergency"
16	"emergency training program*"
17	"emergency medical care"
18	#7 or #8 or #9 or #10 or #11 or #12 or #13 or #14 or #15 or #16 or #17
19	#6 and #18
20	MeSH descriptor: [Personnel Loyalty] this term only
21	MeSH descriptor: [Work Schedule Tolerance] this term only
22	MeSH descriptor: [Work-Life Balance] this term only
23	MeSH descriptor: [Workload] this term only
24	MeSH descriptor: [Personnel Turnover] this term only
25	MeSH descriptor: [Burnout, Professional] this term only
26	MeSH descriptor: [Occupational Stress] explode all trees
27	MeSH descriptor: [Career Choice] this term only
28	MeSH descriptor: [Career Mobility] this term only
29	workforce or manpower or staffing or retention or "work-life balance" or turnover or "leaving medicine" or exiting or burnout
30	career near/4 (choice or mobility or progress* or ladder or promotion or advancement or satisfaction)
31	#20 or #21 or #22 or #23 or #24 or #25 or #26 or #27 or #28 or #29 or #30
32	#19 and #31

HMIC

	Search Term
1	medical staff/
2	exp Paediatricians/
3	(physician$ or doctor$ or trainee$ or foundation year or fy1 or fy2 or sho or shos or senior house officer$ or registrar$1 or staff grade or associate specialist$ or consultant$).mp. [mp=title, other title, abstract, heading words]
4	p?ediatrician$.mp
5	(medical practitioner$ or clinician$).mp.
6	or/1-5
7	emergency services/ or emergency hospitals
8	exp emergency health services/ or accident & emergency services/
9	trauma centres/
10	accident & emergency departments/
11	(emergency medical services or emergency service$ or trauma center$ or trauma centre$).mp.
12	(emergency medicine or pediatric emergency medicine).mp.
13	(emergency department$ or emergency room or casualty department$ or "A&E").mp.
14	“accident and emergency".mp.
15	emergency training program$.mp.
16	emergency medical care.mp.
17	or/7-16
18	6 and 17
19	workforce/ or working hours/ or night work/ or shift work/ or unsocial hours/ or staff turnover/ or working conditions/ or working environment/ or occupational stress/ (8095)
20	occupational choice/ or occupational mobility/
21	(workforce or manpower or staffing or retention or work-life balance or turnover or leaving medicine or exiting or burnout or working conditions or job enrichment or quality of work life or workload or work-related illness$).mp.
22	(career adj4 (choice or mobility or progress$ or ladder or promotion or advancement or satisfaction)).mp
23	((length or shift$) adj2 (work or working)).mp.
24	(working hours or unsocial hours).mp.
25	or/19-24
26	18 and 25

PsychINFO

	Search Term
1	exp physicians/
2	(physician$ or doctor$ or trainee$ or foundation year or fy1 or fy2 or sho or shos or senior house officer$ or registrar$1 or staff grade or associate specialist$ or consultant$).mp. [mp=title, abstract, heading word, table of contents, key concepts, original title, tests & measures]
3	p?ediatrician$.mp.
4	medical practitioner$ or clinician$).mp.
5	or/1-4
6	emergency services/
7	(emergency medical services or emergency service or trauma center$ or trauma centre$).mp.
8	(emergency medicine or pediatric emergency medicine).mp.
9	(emergency department$ or emergency room or casualty department$ or "A&E").mp.
10	accident and emergency".mp.
11	emergency training program$.mp
12	emergency medical care.mp.
13	or/6-12
14	5 and 13
15	workforce/ or work-life balance/ or workload/ or employee turnover/ or exp working conditions/ or job enrichment/ or work rest cycles/ or work week length/ or workday shifts/ or person environment fit/ or "quality of work life"/ or workload/ or work related illnesses/
16	exp occupational stress/
17	career change/ or job satisfaction/ or occupational aspirations/ or occupational choice/
18	occupational mobility/
19	(workforce or manpower or staffing or retention or work-life balance or turnover or leaving medicine or exiting or burnout or working conditions or job enrichment or quality of work life or workload or work-related illness$).mp.
20	(career adj4 (choice or mobility or progress$ or ladder or promotion or advancement or satisfaction)).mp.
21	((length or shift$) adj2 (work or working)).mp.
22	or/15-21
23	14 and 22

Business Source Complete

S1	career choice or career decision or career selection or occupation or workforce or manpower or staffing or retention or work-life balance or turnover or leaving medicine or exiting or burnout or career mobility or career choice
S2	(physician or doctor or medical professional) OR paediatrician OR (medical practitioner or clinician) OR emergency physician
S3	emergency training program$ OR emergency medical care OR emergency health service OR hospital emergency services OR emergency medicine OR pediatric emergency medicine OR (emergency service or emergency medical services or trauma centre) OR (emergency department or emergency room or accident and emergency or accident & emergency or a&e or a & e)
S4	(emergency training program$ OR emergency medical care OR emergency health service OR hospital emergency services OR emergency medicine) AND (S1 AND S2 AND S3)

ProQuest Business Premium Collection

(pub(career choice OR career decision OR career selection OR occupation OR workforce OR manpower OR staffing OR retention OR work-life balance OR turnover OR leaving medicine OR exiting OR burnout OR career mobility OR career choice) OR ab(career choice OR career decision OR career selection OR occupation OR workforce OR manpower OR staffing OR retention OR work-life balance OR turnover OR leaving medicine OR exiting OR burnout OR career mobility OR career choice)) AND (pub(physician OR doctor OR medical professional OR paediatrician OR medical practitioner OR clinician OR emergency physician) OR ab(physician OR doctor OR medical professional OR paediatrician OR medical practitioner OR clinician OR emergency physician)) AND (pub(emergency training program OR emergency medical care OR emergency health service OR hospital emergency services OR emergency medicine OR pediatric emergency medicine OR emergency service OR emergency medical services OR trauma centre OR emergency department OR emergency room OR accident AND emergency OR accident emergency OR a e OR a e) OR ab(emergency training program OR emergency medical care OR emergency health service OR hospital emergency services OR emergency medicine OR pediatric emergency medicine OR emergency service OR emergency medical services OR trauma centre OR emergency department OR emergency room OR accident AND emergency OR accident emergency OR a e OR a e))

Emerald Insight

[Anywhere: retention] AND [Anywhere: doctors] AND [Anywhere: emergency medicine]
