# Supplementary material for: Retention of doctors in emergency medicine: a scoping review of the academic literature
Source: Emerg Med J. 2021 Jun 3;38(9):663–72. doi: 10.1136/emermed-2020-210450 (PMC8380914; doi:10.1136/emermed-2020-210450)
Supplement: Supplementary data [file emermed-2020-210450supp003.pdf]

**Author, Year Journal Origin**	**Title**	**Synopsis**	**Rationale for exclusion**
Aiello and Mellor, 2019<br>J of Integrated Care<br>UK (England)	Integrating health and care in the<br>21st century workforce	Describes a series of case studies demonstrating how NHS organisations have adopted integrated workforce models at scale.	The abstract did not specify the areas of practice. The paper did not contain specific example from EM.
Anwar, 1983<br>Ann Emerg Med<br>USA	A longitudinal study of Residency trained emergency physicians	Questionnaire study of graduates of US emergency medicine training. Asked about background and demographic characteristics, current practice environment and position, income, clinical and academic responsibilities, career goals, commitment, and job satisfaction.	Focuses on practice demographics and, despite abstract stating the data is analysed with attention to long-term commitment to EM, a single question about career goals does not merit inclusion in the review.
Anwar and Hogan, 1979<br>JACEP<br>USA	Residency-trained emergency physicians: where have all the flowers gone?	Questionnaire of newly residency trained EM physicians at the start of the specialty in the US.	Useful historical context, states some anticipated threats to retention but no specifics.
Aziz et al., 2018<br>J Coll Physicians Surg Pak<br>Qatar	Female physicians suffer a higher burnout rate: A 10-year experience of the Qatar EM residency training programme	Questionnaire of EPs who have recently finished training in Qatar focusing on burnout.	Asks reasons for leaving for those who have left, but nothing on retention or sustainability.
Bragard et al., 2015<br>BMC Res Notes<br>Canada	Quality of work life of rural emergency department nurses and physicians: a pilot study	Questionnaire looking at quality of work life of EPs and ED nurses in rural Canada. Pilot study.	Essentially a discussion of the methodological factors that the authors will need to attend to scale up from a pilot study to a larger one.

Branie, 2007<br>J of Health Org and Mgt<br>UK	Part-time work and job sharing in health care: is the NHS a family-friendly employer?	Interviews and questionnaires of managers and workers doing part-time work and job shares.	Not specific to EM.
Chan et al., 2014<br>Hong Kong J. Emerg Med<br>Hong Kong	Emergency physician job satisfaction in Hong Kong	Questionnaire of Hong Kong EPs measuring job satisfaction and intent to leave.	Focus is on intent to leave current workplace not the specialty or profession.
Chen et al., 2017<br>Tzu Chi Medical Journal<br>Taiwan	A survey of the perception of well-being among emergency physicians in Taiwan	Measures self-rated wellness of EPs in Taiwan.	Self-rated measure of intent to leave relates to workplace not profession.
Doan-Wiggins et al., 1995<br>Acad Emerg Med<br>USA	Practice satisfaction, occupational<br>stress, and attrition of emergency physicians	Questionnaire sent to members of ABEM about self-rated job satisfaction, burnout and intention to quit.	Gives estimates for intent to leave and burnout but superficial and does not really address retention or sustainability.
Elder et al., 2020<br>Int Emerg Nurs<br>Australia	The demoralisation of nurses and medical doctors working in the emergency department: A qualitative descriptive study	Descriptive qualitative interviews with 6 EPs and 6 ED nurses. Thematic analysis of transcripts.	While there is a discussion about coping strategies, the focus is how they cope with the stressors rather than how they use them to facilitate career sustainability.
Flynn, 2013<br>Ann Emerg Med<br>USA	Emergency care crisis in the United Kingdom and Ireland: Emergency physician exodus looms in wake of pay cuts, staffing shortages	News article summarising the staffing problem in the UK and. Ireland for an international audience.	News item, useful background only.

Gallery et al., 1992<br>Ann Emerg Med<br>USA	A study of occupational stress and depression among emergency physicians	Questionnaire measuring multiple different psychological scales and intention to leave.	Gives baseline figures for a population but no interrogation for themes or correlation.
Ginde et al., 2010<br>Ann Emerg Med<br>USA	Attrition from emergency medicine clinical practice in the United States	Measures rate of attrition from US EM practice and destination of those leaving.	While rates of attrition are useful, they do not inform what drives retention.
Gregov et al., 2011<br>Croat Med J<br>Croatia	Stress among Croatian physicians: comparison between physicians working in emergency medical service and health centers: pilot study.	Questionnaire about stress levels for those working in different conditions.	Mostly sampled general practitioners.
Kalynych et al., 2011<br>Acad Emerg Med<br>USA	Application of margin in life theory to remediation and attrition rates among emergency medicine residents	Abstract for the Society of Academic Emergency Medicine 2011 Annual Meeting. Study aimed to correlate margin in life scores with attrition.	Conference abstract. Further searching identified doctoral dissertation on which the conference abstract was based which was included (Kalynych 2010).
Klasner, 2011<br>J Investig Med<br>USA	Attrition rates of pediatric emergency medicine fellowship graduates	Survey elucidating the proportion of those who had completed a specific paediatric EM fellowship who still practice paediatric EM.	No data beyond estimated attrition rate.
Krywko et al., 2018<br>Acad Emerg Med<br>USA	Developing a formalized wellness and professional development continuing medical education program for emergency medicine physicians	Faculty are interested in wellness as part of professional development.	No direct link to retention.
Lall et al., 2017	Burnout in board certified emergency medicine physicians	Secondary analysis of the 2014 American Board of Emergency Medicine Longitudinal Survey of EPs	Conference abstract only. Manuscript submitted for publication but not

Acad Emerg Med<br>USA		looking for factors associated with seriously considering leaving the specialty. Age and self-reported burnout associated with higher intention to quit, career satisfaction and a higher ratio of academic writing or administration time lower intention to quit.	available in time to include in the scoping review.
Lee et al., 2013<br>Emerg Med J<br>Taiwan	High risk of 'failure' among emergency physicians compared with other specialists: a nationwide cohort study	Cohort study comparing EPs, surgeons and radiologists using national level data to examine rates of leaving the profession. Higher probability of EPs leaving the profession compared with other specialties in Taiwan.	No data beyond estimated attrition rate.
Meurer et al., 2010<br>Acad Emerg Med<br>USA	The incidence of emergency physician turnover: A prospective cohort study within the INSTINCT trial	Extracts turnover data for EP from administrative data helped to facilitate the running of a clinical trial.	Conference abstract. Measures turnover only. Subsequent paper focuses on this from the context of recruiting to clinical trials.
O'Connor and O'Connor, 2015<br>Emerg Med Australas<br>Ireland	EM training in Ireland: are we future proof?	Review of changes to specialist training in EM in Ireland and explore barriers to sustainable, rewarding careers in the speciality.	Conference abstract. Unable to obtain full report from the authors.
Phillips 2016<br>Emerg Med Australas<br>Australia	A call for ACEM to act on gender inequity in our training programme: A male perspective.	Perspective of a male trainee on gender inequality in EM in Australia.	Fleeting mention of violence as a threat to retention only link to inclusion criteria.
Reiter et al. 2016<br>J Emerg Med<br>USA	The Emergency Medicine workforce: Profile and projections.	Discusses the current and projected EM workforce in the US context.	Neglects retention as a key aspect.

Schneider and Weigl, 2018<br>PLoS ONE<br>Germany	Associations between psychosocial work factors and provider mental well-being in emergency departments: A systematic review.	Demonstrates links between certain work factors and well-being.	No direct link to retention or sustainability made in the paper.
Smith-Coggins et al., 2014<br>J Emerg Med<br>USA	Night shifts in emergency medicine: the American Board of Emergency Medicine longitudinal study of emergency physicians.	Questionnaire looking at the impact of night shifts. EPs report that night shifts negatively impact their health.	Limited to a single question asking if nights shifts had caused them to think about leaving EM.
Thiemt, 2016<br>Emerg Med Australas<br>Australia	A call for ACEM to act on gender inequity in our training programme: A female perspective.	Perspective of a female trainee on gender inequality in EM in Australia.	Alludes to factors related to retention but does not directly address any.
Thomas et al., 1991<br>Ann Emerg Med<br>USA	Faculty attrition among three specialties.	Questionnaire. Sent to departmental chairs of ED that had EM trainees and Cardiology and Orthopaedic chairs at the same hospital. Asked who left and what they went to do. Interviewed those who left to try and find out why. Rates of attrition similar across specialties.	Most who left a department didn't leave the specialty. Excluded as solely focused on rates of attrition.
Van der Goot et al., 2020<br>Med Ed<br>Netherlands	Trainee-environment interactions that stimulate motivation: A rich pictures study	Qualitative interviews with 15 junior doctors using the rich pictures drawing method as a visual tool to capture the complexities of the working environment.	No EPs.
van Schothorst et al., 2017<br>Eur J Emerg Med<br>Netherlands	The role of emergency physicians in the institutionalization of emergency medicine.	Ethnography of looking at institutional work in EM in the Netherlands. Institutional work by EM physicians and other shapes the work domain.	Excluded as no direct link to retention in the article.

Vermare and Frappe, 2012<br>Ann Fr Med d'Ugence<br>France	Career cessation in emergency medicine<br>Abandons de carrières en médecine d'urgence	Questionnaire. Sent to all EM physicians working in a region of France. 43 questionnaires returned. Between 2000 and 2010 10 of the 43 respondents left EM.	Focus on leaving, nil on retention.
Weigl and Schneider, 2017<br>Int Emerg Nurs<br>Germany	Associations of work characteristics, employee strain and self-perceived quality of care in Emergency Departments: A cross-sectional study.	Questionnaire linking work characteristics with work strain.	Focus on doctors from specialties other than EM working in the ED, for example general practitioners or orthopaedic surgeons. No direct link to retention.
Weyman et al., 2019<br>Int J Workplace Heal Man<br>UK	One-way pendulum?<br>Staff retention in the NHS: determining the relative salience of recognised drivers of early exit	Focus group defining reasons for leaving the UKs NHS, large survey to rank these in terms of importance. Not clear than any EPs are included in the sample.	Authors offered to review dataset to see if any EPs conducted the survey but unable to at time of writing due to COVID-19.
Wiley et al., 2002<br>Pediatr Emerg Care<br>USA	A comparison of pediatric emergency medicine and general emergency medicine physicians' practice patterns: Results from the Future of Pediatric Education II Survey of Sections Project	Questionnaire of EM an PEM physicians.	One item was intention to leave but no other direct relevance.
Zun, 1996<br>Acad Emerg Med<br>USA	Emergency Physician Terminations: Doing It Right	The paper focuses on dismissal of emergency physicians from a management perspective.	While it has some strategies to avoid getting to that point, dismissal in this context is from an employer, not from the profession.

EM = emergency medicine

BMJ Publishing Group Limited (BMJ) disclaims all liability and responsibility arising from any reliance placed on this supplemental material which has been supplied by the author(s)

EP = emergency physician
