# Supplementary material for: Retention of doctors in emergency medicine: a scoping review of the academic literature
Source: Emerg Med J. 2021 Jun 3;38(9):663–72. doi: 10.1136/emermed-2020-210450 (PMC8380914; doi:10.1136/emermed-2020-210450)
Supplement: Supplementary data [file emermed-2020-210450supp004.pdf]

Paper Details		Study Methods			
Author<br>Year<br>Journal<br>Origin	Title	Data Collection<br>Method/s<br>Scales/questions	Population	Approach to Analysis	Major Limitations
Estryn-Behar<br><br>2011<br><br>Emerg Med J<br><br>France	Emergency physicians accumulate more stress factors than other physicians- results from the French SESMAT study	Questionnaire.<br>Online.<br>Copenhagen Burnout Inventory.<br>Intention to leave (ITL) the profession with a single question.<br>Work-family conflict scale.<br>Satisfaction with pay scale.<br>Job satisfaction scale.<br>Copenhagen Psychological Questionnaire (COPSOQ).<br>Modified Nurses Early Exit (NEXT) questionnaire.	Physicians working in France on a salaried basis. Available on-line a promoted by 2 major associations of physicians.<br>3196 physicians completed the survey, 538 EPs. The EPs were matched to 1924 physicians by demographic characteristics from the total study population.<br>Available March 28th of 2007 and April 30th of 2008.	Bivariate analyses, using Pearson's Chi-square test, to determine the association of predictors with burnout and ITL.<br>Multivariate analyses of factors linked with burnout and ITL.	Self-reported measures.<br>Single question assessing ITL.<br>Response bias.
Feitosa-Filho et al.<br><br>2017<br><br>Rev Assoc Med Bras<br><br>Brazil	Characteristics of training and motivation of physicians working in emergency medicine	Questionnaire.<br>Medical students applied it to participants.<br>Author developed.<br>General characteristics of workplace and participating EP. Main reasons for working in EM. Degree of satisfaction. Main reasons they might leave.	Physicians working in EDs of medium to large hospitals in a large Brazilian city.<br>24 of 25 possible sites participated.<br>659 EPs participated, approx. 75% of those eligible.<br>Conducted January to March 2012.	Descriptive statistics - means and standard deviation or interquartile range and absolute values and percentages.<br>Chi-square test was used for categorical variables and the Mann-Whitney and Kruskal-Wallis for continuous variables.	The authors interchange 'interview' and 'questionnaire'.<br>Unvalidated questionnaire.
Fitzgerald et al.	The psychological health and well-	Interviews<br>Conducted by the lead	Opportunistic sample of 18 EM consultants in	Interpretive phenomenological analysis (IPA). Involved multiple	Selection bias on multiple levels.

2017<br><br>Emerg Med J<br><br>UK	being of emergency medicine consultants in the UK	author (clinical psychologist).<br>Semi-structured, flexible and non-directive, open questions covering: stressors and challenges at work, relation to psychological health, experiences of coping in these circumstances and the implications of these experiences on their working career.	southwest England.<br>From 5 of 19 eligible sites. The 5 sites had 33 whole time equivalent EPs.<br>Between May and October 2013.	readings of transcripts focusing on the language used and semantic content, explanation, key words and phrases. Grouped into themes exemplified by specific quotations. Important contradictions highlighted. Three level validation. Fellow researcher checked themes were grounded in the data, 2 phycologists checked consistency by analysing a sample transcript, 2 participants checked the analysis for representativeness.	Consultants working full time only, geographically limited.
Goldberg et al.<br><br>1996<br><br>Acad Emerg Med<br><br>USA	Burnout and Its Correlates in Emergency Physicians: Four Years' Experience with a Wellness Booth	Questionnaire Administered at a wellness booth at a conference.<br>Maslach Burnout Inventory and a 79-item questionnaire related to demographics, practice characteristics, including intent to practice EM in the future, and health habits.	Opportunistic sample of 1272 EP attending an annual conference in the USA between 1992 to 1995.	Composite indicators generated from survey responses.<br>Relationship between indicators and moderate/high burnout group and low burnout group compared with chi-squared test.<br>Correlation of each independent variable with raw burnout score determined followed by stepwise logistic regression analysis to rank them. Intercorrelations between significant predictor variables in the multivariate analysis were then examined.	Statistical analysis does not control for multiple comparisons. Selection bias – attendees at a scientific conference who opt to attend the wellness both and complete the questionnaire likely to be different from the wider population of EPs. Self-reported measures of attitudes at a single time point.
Hall et al<br><br>1992<br><br>Ann Emerg Med<br><br>USA	Factors Associated with Career Longevity in Residency-Trained Emergency Physicians	Questionnaire.<br>Postal.<br>Personal demographics, training history, professional demographics, the instrument asked those who left EM to use a three-point scale to rate the importance of aspects of practice in	US EPs who finished training between 1978 and 1982. 539 responses from a population of 858 EPs. 62.8% response rate.	Chi-square and Fisher's exact t to test the differences between responders and non-responders. Fisher's exact t test was used if the number of expected responses in any given group was less than five. Actuarial-method life-table analysis was used to determine the survival rate of EPs. Logistic regression was	Large number of variables applied to the small group who left risks non-existent correlations appearing by chance.<br>Respondent bias.<br>Limited to early career EPs entering the profession though residency training.

		making this decision.		used to compare those who left EM with those who remained for personal and professional demographics.	
Hall and Wakeman<br><br>1999<br><br>J Emerg Med<br><br>USA	Residency-Trained Emergency Physicians: Their Demographics, Practice Evolution, and Attrition from Emergency Medicine	Questionnaire<br>Postal.<br>Updated version of Hall et al. 1992 above.<br>Personal and professional demographics, practice patterns and duties, attrition rate, and reasons for leaving EM.	US EPs who finished training between 1978 and 1988. 1638 responses from a population of 2874 EPs. 58.3% response rate.	Chi-square and Fisher's exact t-test were used to test the differences between responders and non-responders. Summary statistics were used to evaluate the practice patterns of EPs. Kaplan–Meier life table analysis was used to determine the survival rate of EPs within EM. Logistic regression was used to compare those who left EM with those who remained for personal and professional demographics.	Ignores non-residency trained EPs. Unable to obtain mail lists for all residency programmes. Response bias.<br>Large number of variables applied to the small group who left risks non-existent correlations appearing by chance.
James and Gerrard<br><br>2017<br><br>Emerg Med J<br><br>UK	Emergency medicine: what keeps me, what might lose me? A narrative study of consultant views in Wales	Semi-structured interviews.<br>Conduced by lead author - intercalating 4th year medical student.<br>Narrative interviews covering what attracted them to the career, barriers they have encountered, if they had considered leaving, and what keeps them there.	10 consultants from 7 Welsh EDs spread across the country. Conducted between February and April 2015.	Interviews voice recorded and professionally transcribed.<br>Analysis utilised an approach from applied policy analysis - familiarisation, identifying a thematic framework,<br>indexing, charting and mapping, and interpretation.	Explored the views of 10 EPs in a specific geography, may not translate to other EDs or EPs.<br>Selection bias.
Kalynych<br><br>2010<br><br>UNF Graduate Theses and Dissertations.<br><br>USA	The Application of Margin in Life Theory in Regard to Attrition and Remediation Among Emergency Medicine Residents	Questionnaire<br>Handed out at training days.<br>Margin in life (psychological theory of adult development) scale (MILS-EM) compared with intention to leave and remediation. EM	273 responses from 452 EM residents enrolled in 10 different training programmes across 9 south-eastern states of the US.	MILS-EM and intention to leave EM, medicine broadly or chance training programme analysed with Frequency Statistics, *t*-test and Wilcoxon Mann Whitney. Other null hypothesis addressed in the thesis have their analytical plans described.	Geographically limited convenience sample.<br>Respondent bias. Self-reported measure of intention to leave may not correlate with actual attrition. Distributed by superiors – risk of coercion.

		modification of a validated instrument.			
Lloyd et al.<br><br>1998<br><br>Acad Emerg Med<br><br>Canada	Predictive Validity of the Emergency Physician and Global Job Satisfaction Instruments	Questionnaire<br>Postal.<br>Current job status.<br>Demographics.<br>Emergency Physician Job Satisfaction (EPJS) and Global Job Satisfaction (GJS) instruments.<br>14 'reasons for leaving' for those who have left.	232 fulltime Canadian EPs who participated in an earlier study were eligible. 209 responded. Response rate of 93.7%.	Descriptive statistics of attrition rate. Demographics and income of those who continue in EM and those who have left compared with Chi-square test. EPJS and GJS analysed using one-way ANOVA with Scheffe's test. Descriptive statistics applied to reasons for leaving.	Small and fixed sample limited by participation in a previous study. Missing data. Test properties, particularly of EPJS, poor.
Pflipsen et al.<br><br>2019<br><br>Ir J Med Sci<br><br>Ireland	Why our doctors are leaving Irish emergency medicine training	Questionnaire.<br>Free text question asking EPs to reflect on their experience of EM training in Ireland.	Sent to all 43 EPs who had left the Irish EM training scheme from 2011 to 2016. Conducted in February 2016. 30 respondents. 71% response rate.	Analytical approach not documented.	None presented by the authors. Small sample, wording of question not presented. No analytical framework for qualitative data.
Takakuwa et al.<br><br>2013<br><br>Acad Med<br><br>USA	A National Survey of Academic Emergency Medicine Leaders on the Physician Workforce and Institutional Workforce and Aging Policies	Questionnaire.<br>Online survey instrument.<br>Demographics of respondent and EPs in their programme including age, how out-of-hours shifts are staffed, the policies, practices and attitudes towards EPs in the last decade of their career.	Sent to 146 identified EM leaders. 78 responses. Response rate 53%. Distributed October 2009.	Univariate descriptive statistics for closed question, along with standard deviations for continuous data. Thematic analysis of open questions. Bivariate comparisons by age, gender, or years as an EM leader with chi-square or Fisher exact test for categorical data.	Low response rate of mostly white men over 55. Unvalidated survey requesting large amounts of data. Only sampled from academic EDs.
Xu et al.<br><br>1994<br><br>Acad Emerg Med	Emergency Medicine Career Change: Associations with Performances in Medical School and	Cohort Study.<br>Routinely collected data.<br>Final year students career intention.<br>Physicians current specialty from the	Doctors graduating from one US medical school from 1978 to 1987. Compared: those who choose EM and stay, those who move into EM	Specialty preference and actual specialty cross tabulated. Categorical variables were compared using nonparametric tests. Continuous variable analysed using ANOVA F-tests.	Results from a small proportion of graduates from a single US medical school. Statistical analysis does not control for multiple

USA	in the First Postgraduate Year and with Indebtedness	American Medical Association Masterfile. Assessment scores from medical school and specialty training. Education dept from the College registrar's office.	and those who leave. Complete career choice data for 1943 graduates. 34 had planned on EM careers. 75 graduates practicing EM.	Post hoc Duncan tests were performed for analyses with significant F-test results.	comparisons. Assumes reliability of routinely collected data.
Xu and Veloski<br><br>Acad Med<br><br>1992<br><br>USA	Factors Influencing Physicians' Decisions to Remain in Emergency Medicine	Questionnaire. Distribution method unclear. Rate on a scale how much 23 different factors influenced them to stay in EM.	Sent to 53 graduates of one US medical school from 1981-1990 who stated EM as their career preference. Thirty-six responses (response rate 68%).	Mean scores of self-devised scale (0 = no influence, 1 = minor positive influence, 4 = major positive influence).	Presentation as brief communication means detail is lacking. Small sample. No rationale for choice of scale or questions.
